# At the Crossroad of Gene Regulation and Genome Organization: Potential Roles for ATP-Dependent Chromatin Remodelers in the Regulation of CTCF-Mediated 3D Architecture

**DOI:** 10.3390/biology10040272

**Published:** 2021-03-27

**Authors:** Aktan Alpsoy, Surbhi Sood, Emily C. Dykhuizen

**Affiliations:** 1 Purdue University Interdisciplinary Life Science Graduate Program (PULSe), Department of Medicinal Chemistry and Molecular Pharmacology, Purdue University, West Lafayette, IN 47907, USA; aalpsoy@purdue.edu (A.A.); sood9@purdue.edu (S.S.); 2 Purdue University Center for Cancer Research, Department of Medicinal Chemistry and Molecular Pharmacology, Purdue University, West Lafayette, IN 47907, USA

**Keywords:** chromatin remodeler, SWI/SNF, INO80, ISWI, CHD, CTCF, cohesin, looping, 3D genome architecture, gene regulation

## Abstract

**Simple Summary:**

The way DNA is packaged in the nucleus of a cell is important for when and how genes are expressed. There are many levels of packaging, and new techniques have revealed that long-range interactions are important for both promoting and restricting the transcription of genes. Some long-range interactions are mediated by physical loops in the genome where, like a rubber band, the ring-shaped cohesin complex loops sections of DNA bound by CCCTC-binding factor (CTCF). Both cohesin and CTCF act on DNA, and increasing evidence indicates that their function is inhibited by nucleosomes bound to the DNA. In this review, we summarize the current knowledge of how individual chromatin remodelers, which utilize ATP to move nucleosomes on DNA, facilitate or inhibit cohesin/CTCF-dependent looping interactions.

**Abstract:**

In higher order organisms, the genome is assembled into a protein-dense structure called chromatin. Chromatin is spatially organized in the nucleus through hierarchical folding, which is tightly regulated both in cycling cells and quiescent cells. Assembly and folding are not one-time events in a cell’s lifetime; rather, they are subject to dynamic shifts to allow changes in transcription, DNA replication, or DNA damage repair. Chromatin is regulated at many levels, and recent tools have permitted the elucidation of specific factors involved in the maintenance and regulation of the three-dimensional (3D) genome organization. In this review/perspective, we aim to cover the potential, but relatively unelucidated, crosstalk between 3D genome architecture and the ATP-dependent chromatin remodelers with a specific focus on how the architectural proteins CTCF and cohesin are regulated by chromatin remodeling.

## 1. Introduction

### 1.1. “Control Tower” of the Cell: Chromatin

From the zygote to fully differentiated adult cells, almost every cell in humans contains the exact same DNA content. However, functionally and phenotypically, a dendritic cell and a neuron are different, and their transcriptional response to external cues are different. Basal and inducible gene expression is regulated in part by the way DNA is organized in the nucleus. In higher order eukaryotic systems, DNA is packed into a protein-dense structure called chromatin, the basic unit of which is called a nucleosome. Nucleosomes consist of an octamer of four types of basic proteins called histones (two copies of each H2A, H2B, H3, and H4); and the ~146 bp of DNA wrapped around this octamer. Another histone protein, H1 is not a part of the nucleosome; however, it plays key roles in connecting nucleosomes and formation of 3D chromatin structure. Further packing into denser structures, nucleosomes form chromatin fibrils and eventually a 3D organization within the nucleus. It was initially thought that genome packaging is utilized primarily as a way to fit the 2 meters of DNA into the limited space of the nucleus and physically protect it from damage. With advances in molecular biology and molecular genetics, the concept that chromatin is passive and protective has been replaced with the more prominent concept that chromatin contributes to dynamic gene regulation.

### 1.2. Gene Expression Regulation at the Level of Chromatin Organization

**Figure 1:**
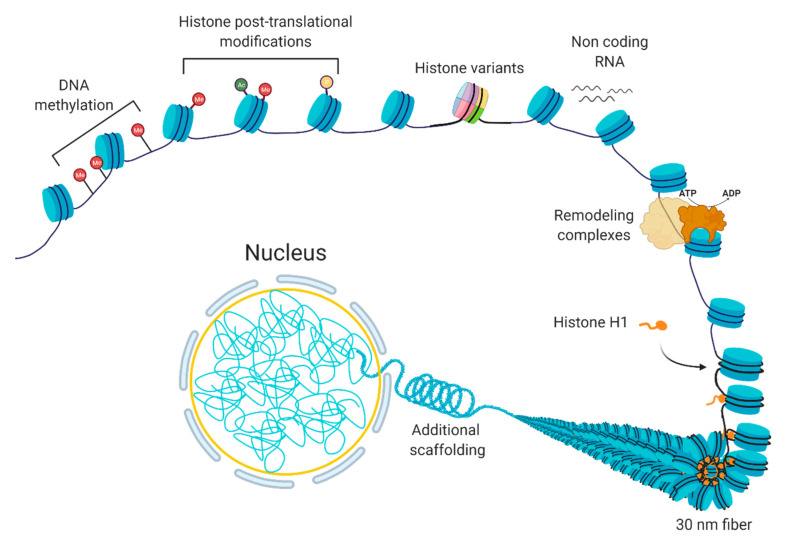
The composition of chromatin. The basic unit consists of DNA and histones, which can be modified or replaced with variants. Additional levels of structural organization rely on chromatin remodeling, ncRNA expression, and scaffolding proteins, such as histone H1.

Chromatin is a dynamic structure that responds to extrinsic and intrinsic cues by facilitating the transcription of appropriate genes. Chromatin can be altered by various processes [1], including DNA methylation, non-coding RNAs, histone variants, histone post-translational modifications, and chromatin remodeling [2,3] (Figure 1). These processes are interrelated; the crosstalk among these various factors coordinate the regulation of the chromatin structure. In basic terms, the probability of genic and regulatory DNA interacting with the transcriptional machinery determines whether a gene is expressed or not. When a chromatin region is densely packed into heterochromatin, the DNA is generally not accessible to the transcription machinery; therefore, it will have little or no transcription. In contrast, when chromatin is more loosely packed into euchromatin, the DNA is accessible to transcription machinery, leading to higher transcription. In normal cells, certain chromatin regions do not change their packing density. For instance, constitutive heterochromatin regions comprise centromeric or telomeric sequences or mobile elements, which are kept silent in most of the normal cell types [4], while housekeeping genes, the genes that are essential for basic cellular functions, tend to be constitutively euchromatic. At other loci, the local chromatin state at specific loci can switch between euchromatin and heterochromatin leading to induction or repression of gene expression.

## 2. 3D Chromatin Organization

The existence of organelles facilitates specialized functions within the cell, much like organs within the body. The compartmentalization within organelles is further used to regulate the local concentrations of proteins, thereby coordinating the interaction between enzymes and their substrates. In the nucleus, the spatial organization of the genome controls the local concentration of transcriptional regulators at genes, which may be equally, if not more, critical for localization than specificity for specific genomic elements.

**Figure 2:**
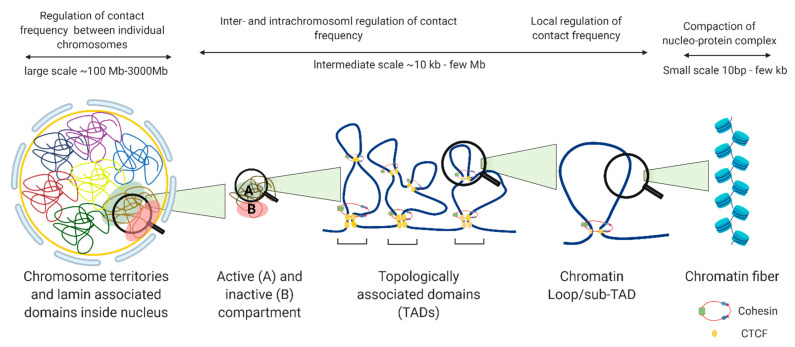
Levels of nuclear genome organization in interphase cells.

The nucleus is organized into large compartments that can be visualized using microscopy, including the nucleolus, speckles, lamina-associated domains, polycomb bodies, and condensates [5,6,7,8]. Advances in next generation sequencing technologies have confirmed these compartmental associations and further revealed that chromosomes tend to cluster into transcriptionally active compartments (“A” compartments) and inactive compartments (“B”) (Figure 2) [9]. Within these compartments are megabase-long topologically associated domains (TADs) that remain mostly consistent across cell types, and can even be conserved between species [10]. Within TADs, genomic regions interact with one another at a higher frequency than with regions outside the TAD, and the maintenance of TAD boundaries is important for restricting transcription to within the boundary. The weakening of TAD boundaries results in pathological chromatin contacts that disrupt gene expression, and this has been associated with diseases such as developmental disorders [11], cancer [12], and other genetic diseases [13]. TADs contain additional levels of compartmentalization in the form of sub-TADs and loops, such as ones that link distal enhancers to promoters and recruit large complexes of proteins such as the mediator complex, preinitiation complex (PIC), and other cell-type specific transcription factors, to transcription start sites [14]. Unlike TADs, enhancer-promoter loops vary significantly between cell types [15], although they are restricted to interactions within TAD boundaries (Figure 2).

**Figure 3:**
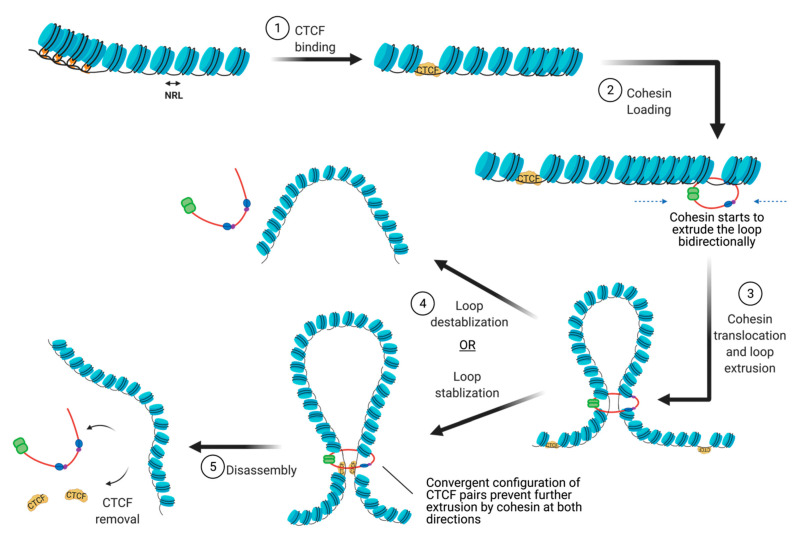
The steps involved in the establishment of CTCF/cohesin mediated looping interactions. (abb: NRL: Nucleosome repeat length).

There are many proteins involved in directing genome organization, such as lamins, which tether the genome to nuclear membranes [16], and polycomb group proteins, which promote the formation of repressive condensates [17,18,19]; however, CTCF and cohesin are the major architectural factors that define both TAD boundaries and sub-TAD loops [10]. CTCF is a ubiquitously expressed zinc finger protein that acts as an architectural protein by binding at a subset of CCCTC consensus sites across the genome [20]. The majority of long-range chromatin interactions contain consensus sequences for CTCF binding which are pointed toward each other in convergent orientation to allow for homodimerization of CTCF [21,22]. Multiple CTCF motifs are enriched at the boundaries of TADs, which restricts the access of cis-acting elements to regions within the TAD boundaries. It performs this function by cooperating with cohesin, a ring-shaped protein complex that physically restrains chromatin into loops at sites where CTCF homodimers are bound [23]. According to the loop extrusion model, a well-accepted model that explains chromatin folding by architectural proteins cohesin and CTCF, the cohesin complex binds the genome and moves along the DNA, forming a growing knot/loop until it reaches CTCF homodimers bound at convergent CTCF motifs [22,24,25]. Several aspects of this process require repositioning of nucleosomes, such as CTCF binding, cohesin loading, cohesin translocation, and cohesin/CTCF eviction (Figure 3). In this review, we focus on recent genome-wide and mechanistic work investigating the roles for chromatin remodeling complexes in regulation of CTCF/cohesin-mediated chromatin interactions.

## 3. ATP-Dependent Chromatin Remodelers

**Figure 4:**
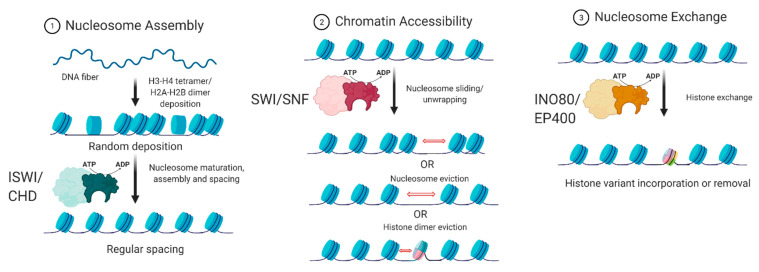
The biochemical functions of ATP-dependent chromatin remodelers. Adapted from [25].

Chromatin remodeling is collectively categorized into three major activities: Assembling the nucleosomes on DNA, altering the physical spacing between the nucleosomes by moving them along DNA or evicting them, and exchanging histone variants [26,27,28]. All activities require hydrolysis of ATP by the ATPase motor, which is utilized to sequentially disrupt (while repositioning) or form (while assembling) histone-DNA contacts. There are four classes of ATP-dependent chromatin remodelers that differ in their structures and activities (Figure 4). Briefly, nucleosome assembly is mostly fulfilled by ISWI and CHD remodelers that mediate random histone deposition and maturation of nucleosomes [26]. Biochemically, ISWI remodelers use a ruler function mechanism to promote nucleosome spacing [29], while CHD remodelers (CHD1-9) help assemble or disassemble nucleosomes. Accessibility of naked DNA is mostly adjusted by SWI/SNF type of remodelers; they can slide nucleosomes along DNA, as well as eject mature octamers or dimers from chromatin. This combined function facilitates the generation of nucleosome-free DNA, which can be recognized by factors that act directly on DNA, such as sequence-specific transcription factors. INO80-type of remodelers are generally associated with incorporation and eviction of histone variants such as H2A.Z whose dynamic exchange is critical during both transcriptional activation and repression, as well as DNA damage repair [26,30]. The direct roles of ATP-dependent chromatin remodelers in the regulation of three-dimensional organization of chromatin have not been studied extensively; however, in recent years, the in-depth analyses of architectural proteins such as CTCF and cohesin have begun to reveal critical functions for ATP-dependent remodelers.

## 4. The Interplay between Chromatin Remodelers and 3D Architectural Proteins

### 4.1. Nucleosome Positioning at CTCF Binding Sites

The nucleosomes around CTCF binding sites are highly positioned or phased, with up to 20 highly organized nucleosomes flanking CTCF, and a nucleosome-depleted region 5′ upstream of the CTCF motif [31,32]. The nucleosome repeat length (NRL; average distance between neighboring nucleosomes) is ~10 bp smaller near CTCF in mouse embryonic stem cells (mESCs), compared to genome-wide average [33]. This difference in nucleosome repeat length at CTCF sites is not due to nucleosome positioning sequencing of the DNA itself, but instead a result of active remodeling [32]. A decreased repeat length is globally observed at active “permissive” chromatin. While this seems counter-intuitive at first, it reflects the removal of linker histones and the partial unraveling of compacted nucleosomes [32,34,35]. A number of ATPase remodelers including BRG1, EP400, CHD1/2/4/8, AND SNF2H overlap with CTCF sites in mESCs and mammary epithelial cells [36]. At CTCF binding sites, CHD1/2/8, EP400, and SNF2H binding sites correlate with decreased NRL [32], although the strongest correlation is observed for SNF2H.

**Figure 5:**
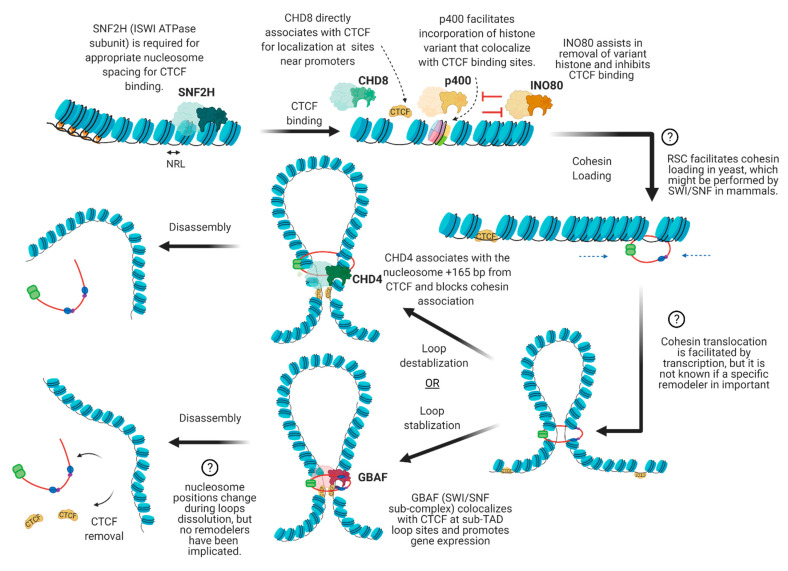
Speculative model illustrating potential roles for remodelers in the establishment of CTCF/cohesin mediated interactions. While the role for SNF2H in nucleosome spacing and CTCF binding is well established, the mechanism by which other remodelers regulate CTCF or cohesin is more speculative. Potential roles based on knowledge of biochemical mechanisms have been proposed, although the chromatin remodeling events may not necessarily involve the modes of chromatin remodeling presented (spacing, variant incorporation, eviction, etc.).

*ISWI:* The ISWI-type ATPase, SNF2H (*SMARCA5*) is the remodeler primarily responsible for the nucleosomal phasing required for CTCF genome binding (Figure 5) [29,32,37]. In HeLa cells, *SNF2H* deletion leads to an increase in nucleosome occupancy and a decrease in nucleosome phasing around CTCF sites [37]. Similarly, in ESCs, depletion of SNF2H, but not BRG1, reduces CTCF binding [29], and reduces the phasing of nucleosomes, that is, the nucleosome position relative to the associated sequence becomes less defined but more fuzzy, and increases the nucleosome repeat length by ~ 9 bp. Further, Hi-C analysis found a reduction in chromatin loops and TADs in the *SNF2H* knockout cells. Likewise, SMC1A (a cohesin complex subunit) Hi-ChIP in *SNF2H*-depleted cells revealed a reduction in cohesin-mediated loops, presumably as a result of decreased CTCF binding.

*CHD8:* Similar to SNF2H, CHD8 binding at CTCF sites correlates with decreased NRL [32]; however, unlike SNF2H, CHD8 is not required for CTCF binding, at least at selected sites [38]. CHD8 directly associates with CTCF, and is dependent on CTCF for localization to selected genomic sites [38], primarily CTCF-sites near transcription start sites [39]. CHD8 is proposed to recruit methyltransferases that affect transcription [38,40]; however, it is not yet clear whether, or how, the ATPase function of CHD8 is involved.

*INO80 subfamily INO80* and *SWR1 (EP400)*: One ATPase in the INO80 subfamily is E1A-interacting protein p400 (or EP400), which can be incorporated into complexes with or without histone acetyltransferase TIP60 [41]. Similar to CHD8, EP400 binding correlates with NRL around the CTCF binding sites [32]; however, the contribution of EP400 to genome organization is not understood. The major activity of p400 is deposition of histone variants H2A.Z and H3.3 into nucleosomes [42,43], making these variant nucleosomes less stable. H2A.Z and H3.3 variants colocalize with CTCF binding sites [31,44,45], forming a permissive chromatin environment for CTCF binding [46]. Additional roles of H2A.Z may include balancing the levels of CTCF deposition and bookmarking the CTCF sites during cell cycle-dependent removal and rebinding of CTCF [47]. Taken together, the evidence suggests that EP400 remodelers may function to enable CTCF localization through H2A.Z deposition (Figure 5). The paralogous remodeler in the subfamily, INO80, performs the opposite reaction, that is, exchanging H2A.Z/H2B dimers with H2A/H2B in yeast [30] or humans [48], in addition to its role in nucleosome sliding [49,50,51]. How that activity may affect CTCF function is not clear. Acting primarily at promoter regions in yeast and mammalian cells [52,53,54], one possible role of INO80 can be counteracting CTCF deposition via H2A.Z removal in a spatio-temporal manner, possibly regulating promoter-enhancer communication. In addition, it was also speculated that INO80 is incapable of cohesin loading in yeast, unlike other types of chromatin remodelers [55], leading to the hypothesis that INO80 may not have direct regulatory roles on architectural proteins.

*SWI/SNF*: The SWI/SNF ATPase BRG1 (*SMARCA4*) was first implicated in 3D genome structure almost 20 years ago, when it was reported to facilitate looping at the alpha-globin and beta-globin loci during erythroid differentiation [56,57,58]. These studies suggested that BRG1 is required for accessibility at the locus control region (LCR), which utilizes looping to coordinate the transcription of specific beta-globin genes. Transcriptional regulation by the LCR is also dependent on CTCF [59] and cohesin [60], although a connection between BRG1 and CTCF/cohesin in the establishment of loops has not been explored. Recently, genome-wide ChIP-seq has identified significant overlap between CTCF and BRG1 binding [61,62,63], while proteomic analysis identifies BRG1 as a direct binding partner of CTCF [64,65]. This implies a potential involvement of SWI/SNF in CTCF function; however, BRG1 binding has little or no correlation to NRL around CTCF [32] and BRG1 deletion has minimal effect on CTCF genomic binding or TAD organization in ESCs [29,32]. In human immortalized mammary epithelial cell line MCF10A, BRG1 depletion resulted in a decrease in the expression of a number of genes, but only a mild effect on TAD organization, as determined with Hi-C. No loss of TADs was observed upon BRG1 knockdown; however, 14% of TAD boundaries were altered such that inter-TAD interactions were increased, and *cis* and *trans* interactions were enhanced at sub-telomeric regions [61]. Cross referencing ChIP-Seq data to BRG1-mediated chromatin occupancy in mouse embryonic fibroblasts (MEFs) indicates that nucleosome occupancy near CTCF sites is reduced upon *BRG1* knockdown [61,62]; however, a mechanistic connection between SWI/SNF-mediated nucleosome remodeling and CTCF/cohesin function has not yet been determined.

One clue that may help is the recent finding that the overlap between CTCF and BRG1 is mediated primarily through the newly characterized GBAF/ncBAF subcomplex, which displays 20-50% overlap with CTCF [66,67,68,69]. CTCF motifs are mainly enriched at GBAF binding sites, while canonical BAF (cBAF) and Polybromo BAF (PBAF) binding sites overlap with other transcription factors [66,67]. Functional assays such as Hi-C or Hi-ChIP focused on the dedicated GBAF subunit BRD9, instead of BRG1, might provide a better picture of SWI/SNF contribution to both short-range and global chromatin interactions, although it is still not clear whether GBAF even has the same remodeling activity in vivo as SNF5-containing SWI/SNF complexes. The questions that remain unanswered are: (1) Does GBAF perturbation cause differential chromatin accessibility or NRL changes? (2) If so, do differentially accessibly regions overlap with CTCF/cohesin binding? Lastly, (3) Does GBAF perturbation affect CTCF/cohesin localization and subsequent loop formation? It is very likely that GBAF is more critical in maintaining the interaction between enhancers and promoters in CTCF-defined transcriptional hubs rather than maintaining TAD boundaries (Figure 5). This may be related to the specific interaction between the GBAF subcomplex with BET proteins BRD2 and BRD4, which are also enriched at CTCF sites, implicated in TAD strength but not boundary establishment [70], and specifically implicated in mediating CTCF-defined promoter-enhancer interactions [71]. Further dissection of the interplay between these factors and cohesin will help resolve how SWI/SNF remodeling facilitates 3D chromatin looping.

### 4.2. Cohesin Loading

Cohesin is a four-subunit protein complex composed of SMC1, SMC3, RAD21, and SCC3, which forms a ring-shaped structure around DNA. According to the loop extrusion hypothesis, cohesin is loaded onto the genome and continues to extrude a longer and longer loop of DNA until it encounters a convergently oriented CTCF homodimerization site [22,72]. At these CTCF sites, cohesin is halted and restrains the chromatin in stable loops, explaining the high overlap between CTCF and cohesin subunits in ChIP-Seq. Recruitment of cohesin to genomic sites in vivo depends on NIPBL and its binding partner MAU2 [73,74,75,76,77]; however, the presence of nucleosomes inhibits both cohesin loading and cohesin diffusion [78], indicating a potential requirement for chromatin remodelers in both processes.

In yeast, cohesin is first loaded to *CEN* sequences at centromeres. These regions are characterized by high “nucleosome fuzziness”, where active nucleosome remodeling results in less defined nucleosome positioning [25]. Yeast RSC chromatin remodeler interacts with cohesin loading complex Scc2-Scc4 and facilitates cohesin deposition [55]. This suggests that in yeast, the remodeling activity of RSC is critical to enable local nucleosome-depleted regions for cohesin binding. In mammalian cells, the cohesin loader subunit NIPBL colocalizes with the transcriptional mediator complex and RNA polymerase II at promoter regions of expressed genes, supporting the idea that cohesin is loaded at nucleosome depleted promoters of actively transcribed genes [79]. Supporting this, more than 50% of NIPBL ChIP-seq peaks are associated with promoters. While SMC3 ChIP-seq peaks overlap with NIPBL ChIP-seq peaks at promoters, a large number of SMC3 peaks are also found at distal regions [80]. CTCF does not overlap with cohesin at the NIPBL binding sites, but at these distal regions near transcribed genes, supporting both the loop extrusion model and evidence that cohesin is loaded primarily at transcribed genes and halted at distal CTCF sites [81]. Cohesin’s role in regulating gene expression at mediator-bound promoters may in fact be through these looping interactions with distal CTCF sites [79,82,83,84]. While specific chromatin remodelers required for cohesin loading in mammals has not been studied extensively, the BRG1 ATPase subunit is required for calcium-induced recruitment of NIPBL to the enhancer regions in activated neutrophils, suggesting a role for SWI/SNF in cohesin loading during activation of immune cells [85].

### 4.3. Cohesin Translocation

Loop extrusion hypothesis dictates that energy is required to propel cohesin along the loop. RNA polymerase II-mediated transcription is one potential energy source, as suggested by the downstream movement of cohesin in response to transcriptional induction at a number of investigated loci [86,87]. The motor for this movement might simply result from transcription-induced supercoiling [78,88], passive diffusion coupled with transcription [89], or other processive enzymes moving along the DNA during transcription [90]. While nucleosome-free regions are required for cohesin translocation [78], these may be indirectly formed as a consequence of nucleosome remodeling for transcription. Despite this established role for transcription in cohesin translocation, acute depletion of RNA polymerase II only alters local short-range contacts, not large-scale genome architecture or TAD structures in mESCs [91]. Therefore, cohesin establishment at TADs may require active recruitment of nucleosome remodelers in a manner independent of the transcriptional machinery.

### 4.4. Cohesin and CTCF Removal

WAPL opens the cohesin ring, resulting in its release from chromatin [92]. Deletion of WAPL in non-replicating cells stabilizes cohesin on chromatin [93], increases average loop size, increases TAD boundary strength, and increases cohesin accumulation at non-convergent CTCF sites [94]. In addition, the expression of many genes decreases, most likely as an indirect effect of depleting the cohesin pool needed for transcription [95] via promoter-enhancer interactions [96]. Single cell dynamics of cohesin binding [97] estimates that >60% of cohesin is dynamic, supporting the notion that cohesin release, as well as binding, is important for transcriptional activity. WAPL association with cohesin is inhibited by cohesin acetylation [98] or cohesin binding with CTCF [99]. While cohesin acetylation has no established relationship with nucleosomes, cohesin-CTCF association is affected by nucleosome positioning. When nucleosomes prevent cohesin from translocating and binding to CTCF, cohesin can be recognized by WAPL and rapidly unloaded from chromatin.

*CHD4:* At CTCF sites co-bound with cohesin, the arrangement of nucleosomes is more symmetrical than at sites without cohesin [32]; however, since the deletion of cohesin subunit *RAD21* has little effect on this nucleosome organization [37] this indicates that a symmetrical arrangement of nucleosomes facilitates cohesin at CTCF sites, and not the other way around. When nucleosomes are organized in an asymmetric manner at CTCF binding sites, there is strong nucleosome occupancy at +105 bp and +165 bp downstream of the motif [32]. Higher nucleosome occupancy, especially at +165, correlates with lower cohesin and RNA polymerase II occupancy. The CHD4 remodeler has the highest genome-wide overlap with CTCF motifs in mESCs [32], but, unlike SNF2H, deletion of CHD4 does not alter the nucleosome repeat length around CTCF binding sites and does not affect CTCF binding to the genome [37]. Instead, CHD4 binding at CTCF sites correlates with a more asymmetric arrangement of nucleosomes, leading to the hypothesis that CHD4 occupancy at CTCF sites promotes a nucleosomal arrangement that is non-conducive to cohesin translocation. Supporting this, CHD4 is directly bound at the restrictive nucleosome +165bp downstream of CTCF (Figure 5). Mechanistically, this explains how recruitment of CHD4 by the transcription factor ETO2 blocks looping between the β-globin locus control region (LCR) to repress γ-globin gene expression during erythroid maturation [100]. In addition, this potential role for CHD4 in promoting cohesin removal through nucleosome positioning is supported by a recent study in granule neurons of mouse cerebellum, which suggests that CHD4 decreases chromatin accessibility, cohesin occupancy, and contacts between enhancers and promoters [101].

### 4.5. Dynamic Regulation of Local Chromatin Interactions upon Stimuli

At TAD boundaries, CTCF binds to more highly conserved consensus sequences [102], binds with greater affinity [32], and maintains binding during differentiation. In contrast, CTCF binding sites at sub-TAD regions and chromatin loops are weaker and more likely to vary between cell types, representing more dynamic chromatin interactions. CTCF sites that are lost during differentiation have shifted nucleosome occupancy patterns, potentially implying that nucleosome remodelers facilitate these changes [32]. Similarly, proinflammatory stimuli can rapidly induce nucleosome remodeling, removal of CTCF/cohesin, and gene expression changes at select loci [103,104], indicating that nucleosome remodelers are required for rapid changes in nucleosome dynamics during the transcriptional response to stimuli. Similar to the finding that cohesin deletion has larger effects on inducible inflammatory gene expression than steady-state gene expression [105], some chromatin remodelers may be more critical in establishing new, or disrupting pre-existing, 3D contacts rather than maintaining them. To our knowledge, there are few investigations of the collaboration between remodelers and architectural proteins in differentiation or acute gene induction models. Individual reports suggest changes in chromatin structure during CTCF binding or relocalization [106,107,108,109] cohesin binding [105,110] or chromatin remodeling [111,112,113,114,115] in response to stimuli, but coordination between these processes and the individual remodelers involved is unexplored.

## 5. Conclusions/Future Directions/Perspective

There is emerging evidence suggesting that various types of ATP-dependent chromatin remodeling complexes facilitate both long-range and short-distance chromatin interactions through regulating the appropriate nucleosome organization for CTCF and cohesin complexes (Figure 5). In some organisms, remodelers have very direct roles, such as in the recruitment of cohesin loading machinery, while in mammalian systems, direct mechanistic roles are less clear. Better understanding of how remodelers directly affect 3D structure will likely require time-resolved studies using directed experimental approaches and tools, such as acute depletion/inhibition of individual remodelers or CRISPR-mediated contact sequence modifications with high-resolution ChIA-PET or Hi-ChIP. Additionally, performing such experiments during stimuli will give insight into the function of remodelers during the assembly/disassembly of loops instead of steady-state maintenance of loops.

## Data Availability

Not applicable.
